# Case report: A study on the pathology of mandibular osteosarcoma with hepatic metastasis in a giant panda

**DOI:** 10.3389/fvets.2025.1542700

**Published:** 2025-02-24

**Authors:** Zongliang Xiong, Shanshan Ling, Caiwu Li, Linhua Deng, Tingting Wei, Ming He, Chengdong Wang, Qihui Luo, Desheng Li, Zhengli Cheng

**Affiliations:** ^1^Experimental Animal Disease Model Research Laboratory, School of Veterinary Medicine, Sichuan Agricultural University, Chengdu, China; ^2^Key Laboratory of State Forestry and Grassland Administration on the Giant Panda, China Conservation and Research Center for the Giant Panda, Chengdu, China

**Keywords:** giant panda, osteosarcoma, tumor metastasis, pathology, cheek osteosarcoma

## Abstract

During routine health examinations, an abnormal growth was detected in the oral cavity of a male giant panda. A malignant tumor, osteosarcoma, was diagnosed through CT (computed tomography) scans and pathological examination of biopsy samples. After two attempts at “tumor reduction surgery” with no improvement, the condition stabilized following particle implantation and arterial infusion interventional therapy. The following year, a CT scan revealed a highly similar mass in the left lumbar muscle, which showed no improvement despite chemotherapy, leading to death 1 month later. Post-mortem examination and tissue pathological diagnosis confirmed osteosarcoma characteristics in the facial, lumbar, and liver masses. The giant panda was diagnosed with osteosarcoma with liver metastasis based on integrated pathological and gross anatomical observations. This case represents the first report of osteosarcoma with liver metastasis in a giant panda, providing valuable data and references for future clinical diagnosis and treatment of tumors in giant pandas.

## Introduction

The giant panda (scientific name *Ailuropoda melanoleuca*) belongs to the bear family, genus Ailuropoda, and has existed on Earth for at least 8 million years. Known as a “living fossil” and the “national treasure of China,” it serves as the emblem of the World Wildlife Fund (WWF) and is a flagship species in global biodiversity conservation. Its disease research is also highly focused. As a severe disease that poses a significant threat to health, the incidence of tumors in giant pandas has correspondingly increased with their extended lifespan. Given the uniqueness of the giant panda, clinical tumor case reports are extremely rare, with only sporadic reports available, such as ovarian cancer, pancreatic ductal adenocarcinoma, seminomas, cutaneous hemangiomas, and conjunctival vascular sarcomas (1–5). This leads to a severe shortage of clinical diagnostic data for tumors in giant pandas, especially lacking in testing data and reports from live sampling.

Osteosarcoma (Osteosarcoma, OS) is a common primary malignant bone tumor (6, 7), and OS is a disease originating from mesenchymal cells, characterized by the proliferation of osteoblast precursor cells and the formation of bone or immature bone (8). It is particularly common in humans and canines (9), and is typically treated with amputation or chemotherapy. Osteosarcoma can arise in any bone, most commonly affecting the long bones of the legs and occasionally the long bones of the arms (10). Osteosarcoma can metastasize from the primary site to other locations, most frequently to the lungs, the same bone, or another bone, which increases the difficulty of treatment and rehabilitation (11–13). This case represents the first report of osteosarcoma in a giant panda and also the first report of osteosarcoma with liver metastasis in a giant panda, filling a gap in the clinical data of giant panda osteosarcoma and holding significant reference value.

## Case presentation

### Basic case information

Giant panda “Wugang” (lineage number 502), a male, was rescued in the wild in 1999. CT scans in May 2021 revealed irregular local bone quality in the right zygomatic arch, with adjacent soft tissue swelling and multiple small nodular calcifications; no other significant abnormalities were observed. In February 2023, an abnormal growth was found in its oral cavity, accompanied by symptoms such as bleeding during feeding. After examination and diagnosis, a malignant tumor, osteosarcoma, was confirmed. Two attempts at tumor reduction surgery were made, but the recovery was poor. Due to the astonishing growth rate of the osteosarcoma, the frequency and volume of bleeding increased, leading to severe anemia in the panda. To better control the tumor and reduce bleeding, Wugang underwent interventional chemotherapy, including particle implantation and arterial infusion, which effectively controlled the bleeding and reversed the anemia, stabilizing the condition for a period. In September 2023, a follow-up enhanced CT scan showed the oral tumor to be relatively stable, but a mass approximately 9.8 cm × 7.7 cm was found in the left lumbar muscle, highly similar in nature to the oral tumor (osteosarcoma), with surrounding bone tissue destruction affecting normal walking. After intravenous chemotherapy, the panda’s condition did not improve and the panda died at the age of 24. For treatment of tumors, please see the Supplementary material.

### Necropsy findings

**Figure 1:**
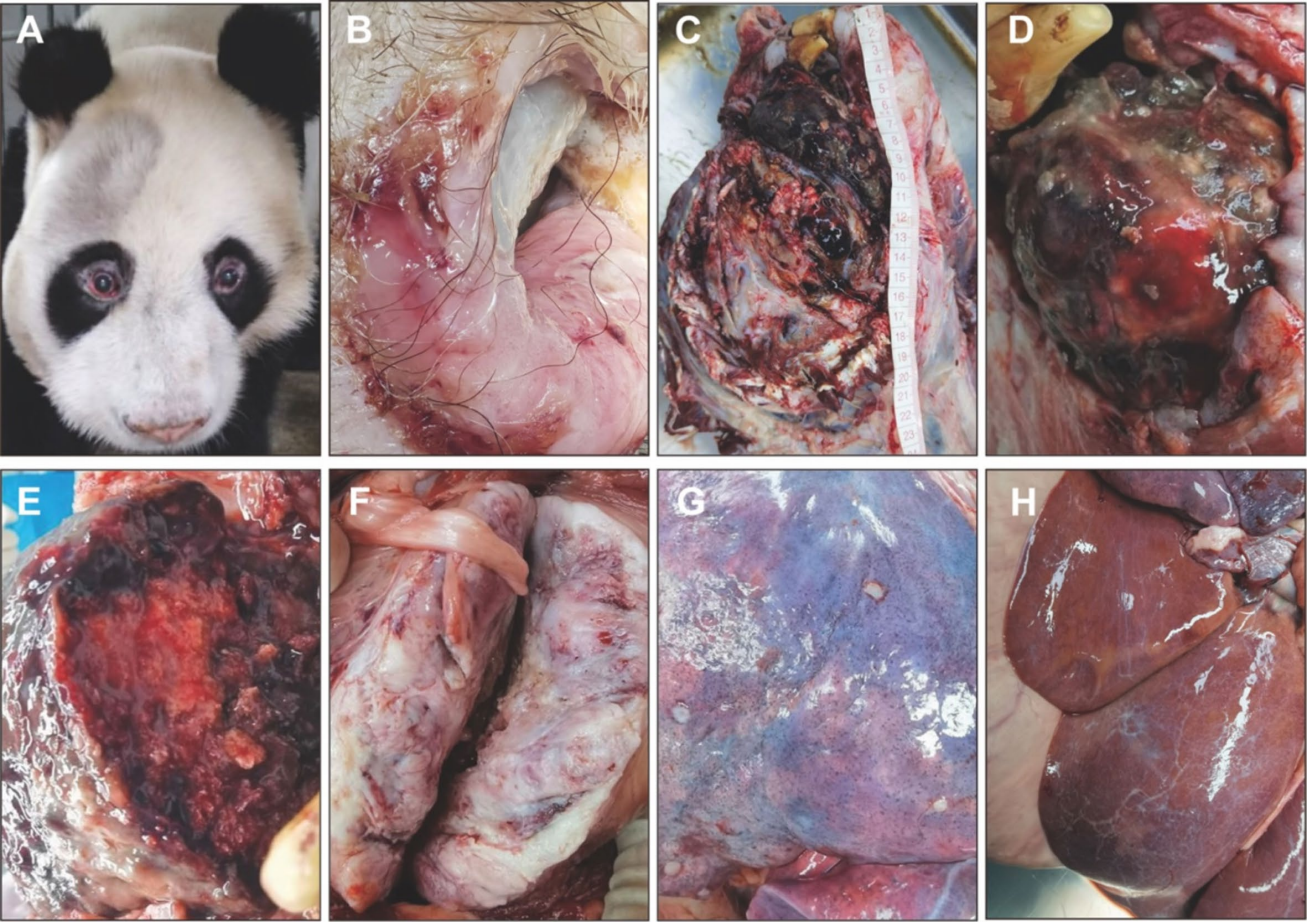
Clinical necropsy examination of giant panda Wugang. **(A)** Facial appearance before death. **(B)** Oral margin. **(C)** Location and size of the cheek mass. **(D)** Surface characteristics of the cheek mass. **(E)** Cross-sectional characteristics of the cheek mass. **(F)** Cross-sectional characteristics of the lumbar mass. **(G)** Nodules on the lung surface. **(H)** Pale yellow foci can be seen in the liver.

The body weight was 86 kg, with a total body length of 112 cm, head length of 37 cm, hind limb length of 59 cm, forelimb length of 65 cm, chest circumference of 106 cm, abdominal circumference of 113 cm, and neck circumference of 71 cm. The cadaver appeared clean, emaciated, with dull fur; the right cheek was swollen, and the mouth was draining brown liquid (Figures 1A,B). Upon necropsy, a large bony mass was found in the right cheek and mandible area, with a surface that exhibited a gradient of color from black to grayish-white, resembling a cauliflower-like variation; the central part of the mass was a pinkish bone structure, and the mass occupied the entire right side of the face (Figures 1C,E). There was a mass measuring approximately 10 cm × 10 cm in the left lumbar muscle area, presenting as a solid structure with varying shades of pink (Figure 1F). The stomach was distended with gas, the gastric wall had pinpoint bleeding, there was hemorrhaging in the peritoneum, congestion in the intestinal walls, hepatic edema, multiple pale gray nodules on the lung surface, each about 0.5 cm in diameter, and the lungs showed mild atrophy and collapse (Figure 1G). The liver is slightly enlarged, lighter in color, and yellowish foci are visible (Figure 1H).

### Imaging examination

**Figure 2:**
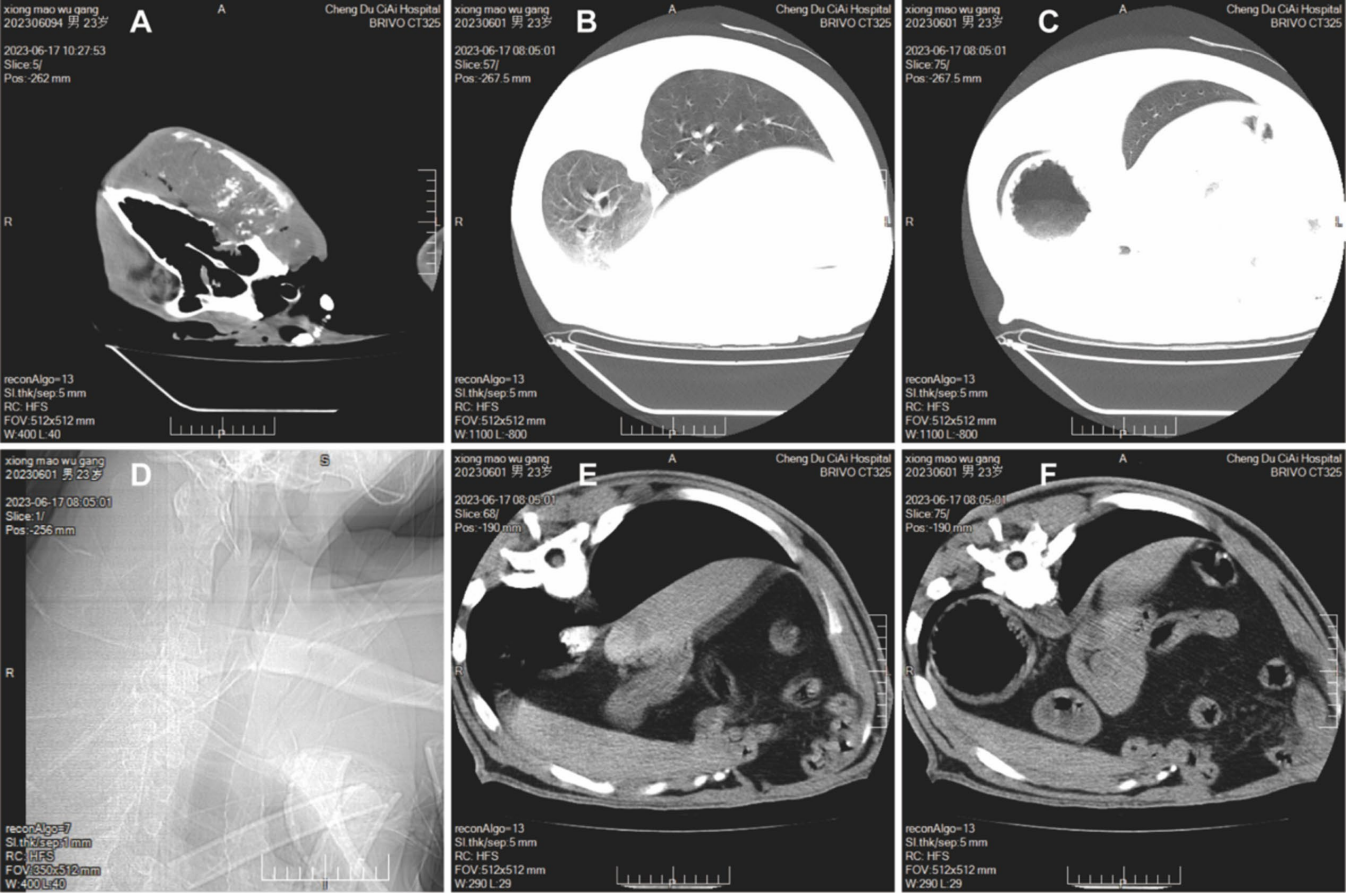
CT scans of giant panda Wugang. **(A)** A mass is visible in the right maxillofacial region. **(B)** A bulla is observed in the left lower lobe of the lung. **(C)** Pleural effusion is present. **(D)** Degenerative changes in the vertebrae. **(E)** Enlargement of the left adrenal gland with multiple calcified foci. **(F)** Swelling of the left psoas major muscle with a visible mass.

CT scans revealed a local mass in the right maxillofacial region extending to the right side of the neck, with the largest dimension measuring approximately 204 × 113 × 124 mm. The lesion had uneven density, with multiple patchy high-density and gas shadows, and showed mild heterogeneous enhancement on contrast scans. The skull and right maxillofacial bones were invaded and destroyed (Figure 2A). CT scans of the thorax and abdomen demonstrated a bulla in the left lower lobe of the lung (Figure 2B); left pleural effusion measuring approximately 20 mm in thickness, and right pleural effusion measuring approximately 10 mm (Figure 2C); degenerative changes in the 12th and 13th thoracic vertebrae and intervertebral discs (Figure 2D); enlargement of the left adrenal gland with multiple calcified foci (Figure 2E); swelling of the left psoas major muscle with an internal mass measuring approximately 98 × 77 mm, uneven density, and patchy non-enhancing shadows. Contrast scans showed peripheral rim enhancement, seemingly with small vessels traversing through, and the internal low-density focus did not enhance, with surrounding bone destruction (Figure 2F).

### Pathological examination

#### Biopsy tissue pathology examination

**Figure 3:**
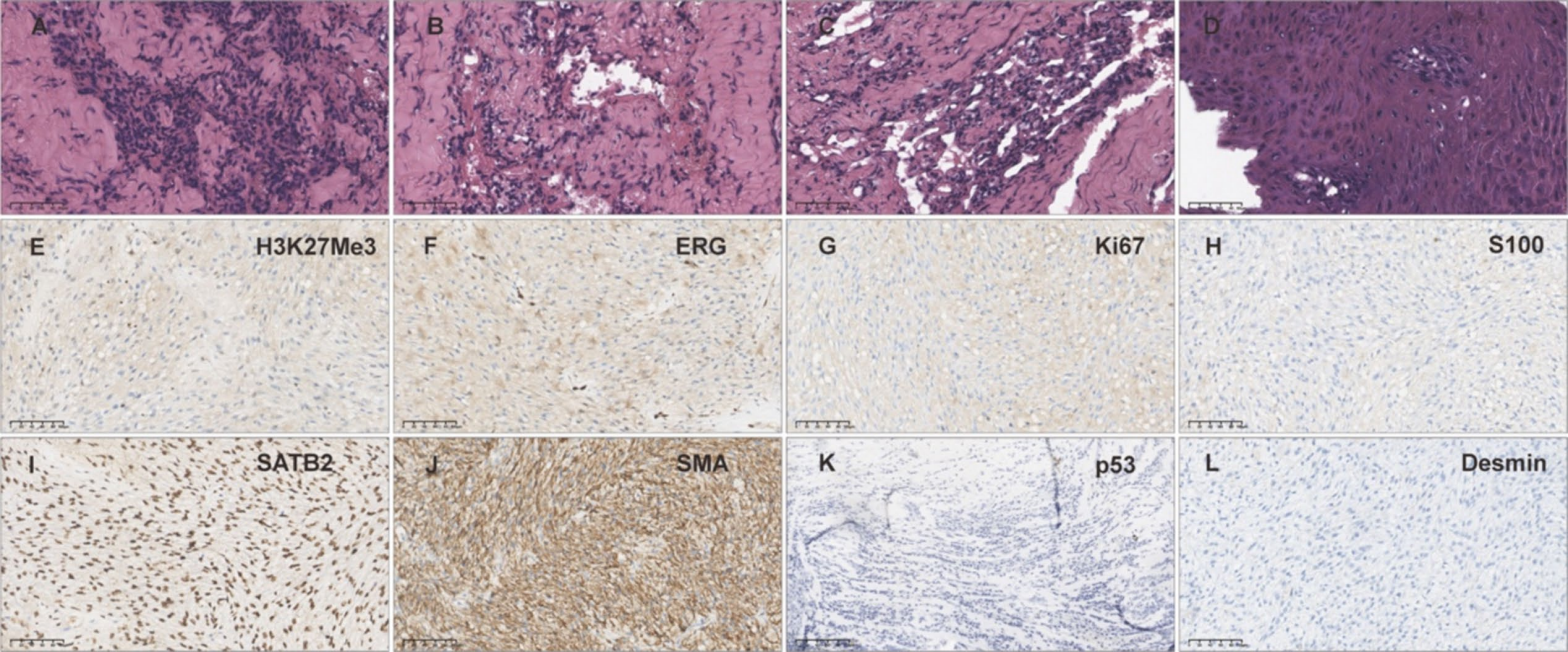
Biopsy tissue HE staining and immunohistochemical examination. **(A–D)** HE staining of the biopsy sample from the cheek mass. **(E)** H3K27Me3 staining of the biopsy sample from the cheek mass (partially lost). **(F)** ERG staining of the biopsy sample from the cheek mass (−). **(G)** Ki67 staining of the biopsy sample from the cheek mass (inconclusive). **(H)** S100 staining of the biopsy sample from the cheek mass (−). **(I)** SATB2 staining of the biopsy sample from the cheek mass (+). **(J)** SMA staining of the biopsy sample from the cheek mass (partially +). **(K)** p53 staining of the biopsy sample from the cheek mass (−). **(L)** Desmin staining of the biopsy sample from the cheek mass (−).

After the excision of the mass from the right cheek, paraffin sections were prepared and HE (Hematoxylin–Eosin)-stained, with a pathological diagnosis of spindle cell tumor, easily visible mitotic figures (Figure 3A), presence of atypical cartilage and tumorous osteogenesis (Figures 3B,C), and the tissue morphology was that of a high-grade malignant tumor (sarcoma), graded as 3 (G3) by the FNCLCC (French Federation of Comprehensive Cancer Centers) system. The subtype diagnosis, combining tissue morphology and immunohistochemical results, was conventional osteosarcoma (fibroblastic subtype). Surface squamous epithelial ulceration with significant hyperplasia and pseudoepitheliomatous hyperplasia (Figure 3D). Immunohistochemical results: tumor cells showed SATB2(+), Desmin(−), S100(−), SMA(partially +), H3K27Me3(partially lost), ERG(−), Ki-67(MIB-1)(inconclusive) (Figures 3E–L).

### Post-mortem tissue pathology examination

**Figure 4:**
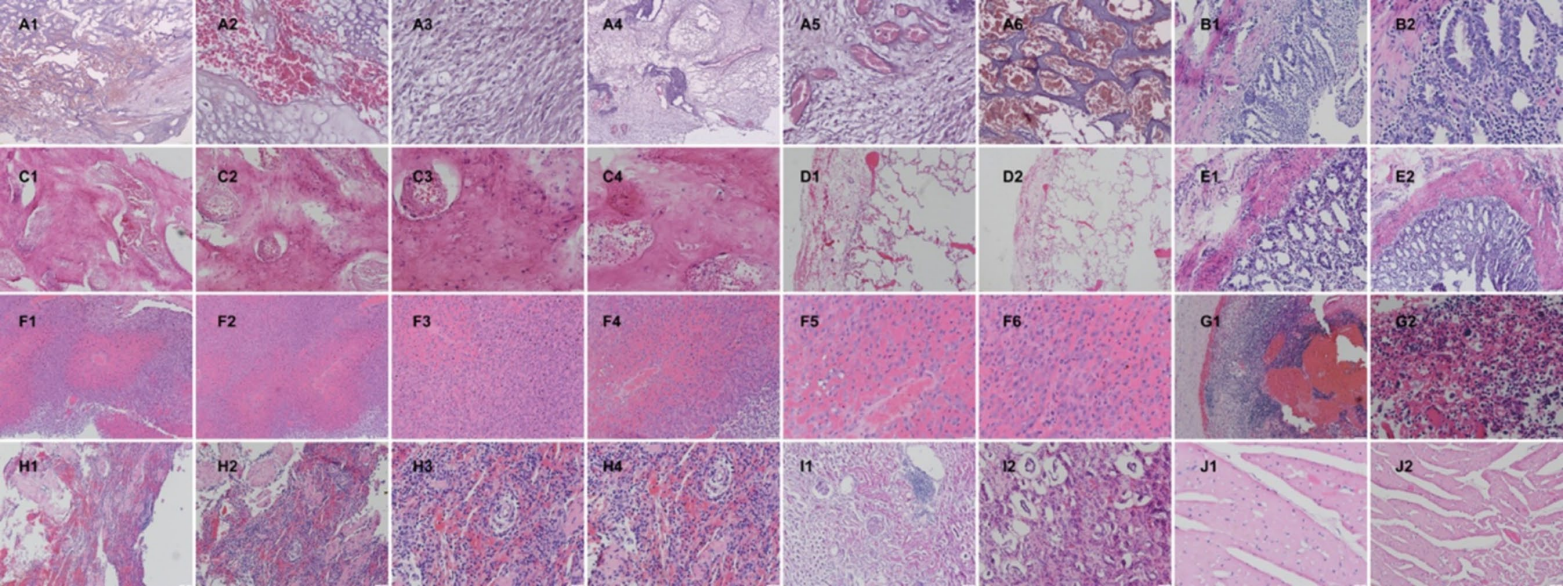
Post-mortem tissue sampling with H&E staining for pathological diagnosis. **(A)** Facial mass, with different Arabic numerals indicating different fields and magnifications, A1 40X, A2-A3 400X, A4 100X, A5 400X, A6 200X. **(B)** Jejunal tissue, B1 200X, B2 400X. **(C)** Mass in the lumbar region, C1 100X, C2 200X, C3-C4 400X. **(D)** Lungs, D1 100X, D2 40X. **(E)** Duodenum, E1 200X, E2 400X. **(F)** Liver, F1 100X, F2-F4 200X, F5-F6 400X. **(G)** Lymph node G1 100X, G2 400X. **(H)** Spleen, H1 100X, H2 200X, H3-H4 400X. **(I)** Kidney, I1 100X, I2 200X. **(J)** Heart, J1 400X, J2 200X.

After necropsy, tissue samples from the major organs of the giant panda were collected, with bone tissues undergoing decalcification treatment and the remaining tissues undergoing routine dehydration and clarification. Paraffin sections were then prepared, stained with HE, and histopathological changes were observed. The results are as follows: Facial mass: The mass tissue is primarily composed of large areas of interlacing neoplastic immature bone or cartilage. There is significant proliferation of fibrous new tissue between the bone plates, accompanied by a large number of blood vessels with vascular congestion. Most cells in the bone plates appear vacuolated. The fibrous tissue exhibits marked cellular pleomorphism, with spindle, oval, or round shapes, and contains a large amount of collagen. Mitotic figures are frequently observed (Figures 4A1–A6). In conjunction with the gross anatomical protruding proliferative mass on the facial surface, a preliminary diagnosis of osteosarcoma is made. Jejunum: Mucosal autolysis/necrosis (Figures 4B1,B2). Lumbar mass: The mass tissue is primarily composed of large areas of interlacing neoplastic immature bone. Between the bone plates, there are mature red blood cells and bone marrow cells, with erythroid hyperplasia. The bone plates contain a high amount of bone collagen, with cellular atypia, round, oval, and spindle shapes, and frequent mitotic figures (Figures 4C1–C4). In conjunction with the gross anatomical protruding proliferative mass, a preliminary diagnosis of osteosarcoma is made. Lungs: The pleura exhibits hyperplasia and hemorrhage. There is alveolar overinflation, rupture of the alveolar septa, and vascular congestion (Figures 4D1,D2). Duodenum: Dilation of intestinal glands, atrophy of intestinal villi, and massive inflammatory cell infiltration (Figures 4E1,E2). Liver: The liver tissue is largely replaced by tumor cells, with hepatocytes being compressed, and the cellular and tissue structure disappearing. The tumor tissue is arranged in sheets, with large cell volume, tight arrangement, obvious atypia, round, oval, and spindle shapes, and a high nuclear-cytoplasmic ratio (Figures 4F1–F6). A preliminary judgment is made for osteosarcoma liver metastasis. Lymph nodes: The lymph nodes show extensive congestion and hemorrhage, tissue structure disorder, almost no mature lymph follicles in the cortical area, a significant reduction in cellular components, and a large number of multinucleated giant cells (Figures 4G1,G2). Spleen: The spleen is atrophied and degenerated, with the structure disappearing, a significant reduction in cellular components, and splenic trabecular hyperplasia (Figures 4H1–H4). Kidneys: Dilation of renal tubules, thinning of tubular walls, and detachment of some renal tubular epithelium. Focal inflammatory cell infiltration is observed (Figures 4I1,I2). Heart: Myocardial cell atrophy, interstitial edema, and brown pigment deposition (Figures 4J1,J2).

## Discussion

Giant pandas, due to their unique characteristics, can survive for several decades in captive environments, compared to their wild habitats, they have a longer lifespan. With the extension of the giant panda’s lifespan, the incidence of tumors also increases, posing a threat to the lives of elderly giant pandas. Currently, there are few clinical reports of tumors in giant pandas, and there is a severe lack of clinical data. This case is the second tumor case diagnosed pathologically in our laboratory, with both cases having tumors located in the oral cavity (accepted), which may be related to the feeding habits of giant pandas. In canine osteosarcoma cases, tumors are commonly located in the limbs, and the treatment often involves removing the tumor mass while trying to preserve the limb, but sometimes, to eradicate all cancerous lesions, part of the limb may need to be amputated (14, 15). In this case, due to the tumor’s location in the maxillofacial region, the treatment methods used in canine osteosarcoma cases could not be applied, and due to the absence of data on giant panda osteosarcoma cases, there were no referable treatment methods. By referring to treatment methods from human medicine, a tumor reduction surgery was first performed, but with poor results; eventually, particle implantation and arterial infusion were used to inhibit further tumor progression.

In canine osteosarcoma cases, osteosarcoma most commonly metastasizes to the lungs, the same bone, or another bone (16, 17). In this case, the maxillofacial osteosarcoma in the giant panda, after being stabilized with interventional treatment, experienced metastasis the following year. However, unlike the spread of canine osteosarcoma, after the stabilization of the maxillofacial osteosarcoma in this case, a mass with the same characteristics as the maxillofacial tumor was observed in the lumbar muscle by CT scan, and post-mortem sampling with H&E staining revealed similar tumor features in the liver tissue. Owing to the scarcity of clinical tumor case data in giant pandas, it is unclear whether this particular pattern of tumor metastasis is an isolated case or a common feature. This case represents the first reported instance of osteosarcoma in giant pandas, establishing a systematic and comprehensive diagnostic approach. The liver metastasis of osteosarcoma in this case also provides valuable reference and clinical data for the treatment of osteosarcoma in giant pandas.

## Data Availability

The original contributions presented in the study are included in the article/Supplementary material, further inquiries can be directed to the corresponding authors.
