# Supplementary material for: Case report: A study on the pathology of mandibular osteosarcoma with hepatic metastasis in a giant panda
Source: Front Vet Sci. 2025 Feb 24;12:1542700. doi: 10.3389/fvets.2025.1542700 (PMC11892105; doi:10.3389/fvets.2025.1542700)
Supplement: Supplementary file 1 [file Table_1.docx]

Supplementary materials

Particle implantation: sealed seed source iodine [125I] 10.1-6.0mCi*, dose: 310 particles.

Arterial perfusion: 60 mg of cisplatin plus 250 ml of normal saline, 40 drops per minute.

Intravenous chemotherapy: 40 mg of epirubicin hydrochloride plus 100 ml of normal saline, intravenous drip, 40 drops per minute. 60 mg of cisplatin plus 250 ml of normal saline, 40 drops per minute.
